# Proinflammatory Differentiation of Macrophages Through Microparticles That Form Immune Complexes Leads to T- and B-Cell Activation in Systemic Autoimmune Diseases

**DOI:** 10.3389/fimmu.2019.02058

**Published:** 2019-08-28

**Authors:** Catalina Burbano, Juan Villar-Vesga, Gloria Vásquez, Carlos Muñoz-Vahos, Mauricio Rojas, Diana Castaño

**Affiliations:** ^1^Grupo de Inmunología Celular e Inmunogenética, Facultad de Medicina, Instituto de Investigaciones Médicas, Universidad de Antioquia UdeA, Medellin, Colombia; ^2^Unidad de Citometría de Flujo, Sede de Investigación Universitaria, Universidad de Antioquia UdeA, Medellin, Colombia; ^3^Sección de Reumatología, Hospital Universitario San Vicente Fundación, Medellin, Colombia

**Keywords:** microparticles, macrophage, M1-like activation, M2-like activation, systemic autoimmune diseases, rheumatoid arthritis, systemic lupus erythematosus

## Abstract

Patients with rheumatoid arthritis (RA) and systemic lupus erythematosus (SLE) demonstrate increased circulating microparticles (MP). These vesicles, primarily those that form immune complexes (MP-IC), may activate monocytes. We evaluated the effect of MP and MP-IC in the differentiation of monocytes to macrophages (monocyte-derived macrophages; MDM) and for consequences in autologous lymphocyte activation. Monocytes from healthy controls (HC) and patients with RA and SLE that differentiated into MDM in the presence of MP-IC showed a proinflammatory (M1-like) profile, which was more evident using MP-IC from patients with RA than those from patients with SLE. Notably, MDM from HC and patients with RA that differentiated with MP-IC were more prone to M1-like profile than those from patients with SLE. In HC and patients with RA, monocyte differentiation using MP-IC decreased the frequency of MDM that bound/internalized latex beads. The M1-like profile did not completely revert following IL-4 treatment. The effect of M1-like MDM on T lymphocytes stimulated with phytohemagglutinin was further evaluated. MDM differentiated with MP enhanced the proliferation of T cells obtained from patients with RA compared with those differentiated with MP-IC or without vesicles. Neither MP nor MP-IC induced interferon (IFN)-γ+ and tumor necrosis factor (TNF)-α+ T cells in patients with RA. Conversely, unlike MDM differentiated with or without MP, MP-IC enhanced the proliferation and increased the frequencies of IFN-γ+CD4+ T, TNF-α+CD4+ T, and IFN-γ+CD8+ T cells in patients with SLE. The co-culture of B cells with MDM obtained from patients with RA and SLE and differentiated with MP-IC increased the expression of B-cell activation markers and prevented B lymphocyte death. Strikingly, only for patients with SLE, these responses seemed to be associated with a significant increase in B-cell activating factor levels, high plasmablast frequency and immunoglobulin production. These results showed that MP-IC from patients with systemic autoimmune diseases favored the polarization of MDM into a proinflammatory profile that promotes T-cell activation, and additionally induced B-cell activation and survival. Therefore, the effect of MP-IC in mononuclear phagocytes may be an important factor for modulating adaptive responses in systemic autoimmune diseases.

## Introduction

Rheumatoid arthritis (RA) and systemic lupus erythematosus (SLE) are chronic systemic autoimmune diseases affecting a large number of people globally (1). The etiology of SLE and RA is not completely known; however, both diseases are characterized by the presence of antibodies against self-antigens (2, 3). These autoantibodies have been associated with a loss of central and peripheral mechanisms of tolerance, as well as with tissue damage and the maintenance of chronic inflammatory responses, through immune complex (IC) formation, recognition and tissue deposits (2, 3). Recently, extracellular vesicles such as microparticles (MP) were reported as one of the main sources of circulating IC in RA and SLE (4–6). Such cell membrane-derived vesicles are primarily formed during cell death and activation and exhibit a broad spectrum of physiological functions, such as intercellular communication, different phases of innate and adaptive immunity, apoptosis, and cellular homeostasis, in healthy individuals (7).

In RA and SLE, alterations in the count, phenotype, recognition, and function of MP and MP forming IC (MP-IC) have been reported (4, 5). MP from patients with RA and SLE reportedly contain proinflammatory components and autoantigens such as citrullinated peptides, high-mobility group protein 1 (HMGB1), and nucleic acids (4, 8). Mononuclear phagocytes, mainly monocytes and macrophages, play critical roles in depurating apoptotic cells, MP and IC (9, 10); interestingly, MP-IC are more efficiently bound and internalized by these phagocytes than MP alone (4). Therefore, MP have been postulated to activate and define the functional profile of monocytes and macrophages obtained from patients with RA and SLE via the activation of Toll-like receptors (TLR) -4, -9, and -7, which recognize oxidized HMGB1, DNA, and RNA, respectively. In addition, MP-IC seem to additionally signal mononuclear phagocytes via Fcγ and complement receptors, thus perpetuating the inflammatory process in these patients (11, 12).

Monocytes and macrophages are key components of the innate immune system and have numerous functions such as phagocytosis, antigenic presentation, and cytokine production (10, 13). In the murine model, many tissue macrophages, such as microglia in the brain, peritoneal macrophages, and Kupffer cells in the liver, originate from the yolk sac, or fetal liver progenitors (14). In adult mice, macrophage populations in the lung, peritoneal cavity, and spleen are more heterogeneous owing to the presence of bone marrow-derived macrophages during steady state and inflammation (14, 15). Under inflammatory conditions, resulting from damaged tissues following an infection or injury, monocytes are recruited from the circulation and are differentiated into monocyte-derived macrophages (MDM) while migrating to the affected tissues (10). MDM often show a proinflammatory phenotype and function, which is primarily attributed to the local inflammatory environment. MDM can secrete various inflammatory mediators, including tumor necrosis factor (TNF)-α, interleukin (IL)-1β, -12, and -6. If the inflammatory response is not quickly controlled, these phagocytes can become pathogenic and contribute to disease progression, as demonstrated in numerous chronic inflammatory and autoimmune diseases including atherosclerosis, multiple sclerosis, inflammatory bowel diseases, RA, and SLE (16–19). The number of macrophages in the synovia and kidneys of patients with RA and SLE correlates with joint damage and glomerulonephritis development, respectively (20, 21), as well as with clinical responses to therapy for both diseases (22, 23).

Reportedly, activated macrophages exhibit significant functional heterogeneity and may polarized into one of two main phenotypes: classically activated M1 (proinflammatory) and alternatively activated M2 (anti-inflammatory) (24). Macrophages stimulated by lipopolysaccharide (LPS) and interferon (IFN)-γ exhibit an M1 profile. As a result, M1 macrophages release high levels of proinflammatory cytokines and chemokines, which in turn promote the recruitment and activation of Th1 and NK cells. M1 macrophages exhibit plasticity and switch their phenotype toward an alternative profile *in situ*. Alternatively, IL-4 induces the production of M2 macrophages that counter inflammation via the phagocytosis of apoptotic neutrophils, production of anti-inflammatory cytokines and increased synthesis of mediators involved in tissue remodeling, angiogenesis and wound repair (25).

Currently, the components of the inflammatory cascade that may favor the perpetuation of the M1 phenotype in macrophages of patients with RA and SLE are still unclear. In this study, we propose that circulating MP, and especially MP-IC, of patients with SLE and RA can directly alter the differentiation of monocytes into proinflammatory macrophages, contributing to the amplification, and perpetuation of autoimmune phenomena and chronic inflammation in affected tissues. The effect of circulating MP and MP-IC obtained from patients with RA and SLE in monocytes was evaluated for their differentiation into macrophages; the functional consequences of this process were also assessed in T- and B-cell activation.

## Materials and Methods

### Reagents, Materials, and Antibodies

RPMI-1640 GlutaMAX medium, Dulbecco's phosphate-buffered saline (DPBS) and fetal bovine serum (FBS) were purchased from Gibco–BRL (Grand Island, NY, USA). Histopaque®-1077, trypan blue, dimethyl sulfoxide anhydrous ≥99.9% (DMSO), tween-20, phytohemagglutinin leukoagglutinin (PHA) and bovine serum albumin (BSA) were obtained from Sigma Aldrich (St. Louis, MO, USA). Penicillin and streptomycin were purchased from Cambrex-BioWhittaker (Walkersville, MD, USA). The BD^TM^ Human Inflammatory Cytometric Bead Array (CBA) was purchased from BD Pharmingen (San Diego, CA, USA). The Rosette Sep Human B cell and T cell Enrichment Cocktails were obtained from STEMCELL Technologies (British Columbia, Vancouver, Canada). Brefeldin A was procured from eBioscience (San Diego, CA, USA). The probes carboxyfluorescein succinimidyl ester (CFSE), LIVE/DEAD Fixable Aqua Dead Cell Stain and FluoSpheres latex beads OR were procured from Invitrogen (San Diego, CA, USA). Recombinant human (rh) CD40 Ligand (CD40L), rhIFN-γ, and rhIL-4 were purchased from R&D Systems; rhIL-2 from Biolegend (San Diego, CA, USA) and the affinity-purified F(ab')_2_ fragment anti-human IgM (anti-BCR) and F(ab')_2_ anti-IgG fragment Alexa Fluor 488 conjugated from Jackson ImmunoResearch (New Baltimore, PA, USA). Monoclonal anti-human MY4 (CD14)-FITC (Clone 322A-1) antibody was obtained from Beckman Coulter; monoclonal antibodies against human CD16-V450 (Clone 3G8), CD32-PE (Clone 3D3), CD36-APC (Clone CB38, also known as NL07), CD3-PerCP (clone SK7), CD4-PE-Cy7 (Clone RPA-T4), CD19-V450 (Clone HIB19), CD69-PE (Clone FN50), CD80-FITC (Clone MOPC-21), CD86-PE-Cy5 (Clone IT2, 2), IFN-γ-APC (clone B27), TNF-α-PE (Clone 6401.1111), CD163-PE (Clone GHI/61), CCR2-Alexa Fluor 647 (Clone 48607), and CD20-PE (Clone L27) were acquired from BD Pharmingen (San Diego, CA, USA). Anti-human HLA-DR-APC-Cy7 (Clone L243), CD209-APC (Clone 9E9A8), CD19-Brilliant Violet 650 (Clone HIB19), CD38-Brilliant Violet 785 (Clone HIT2), CD138-APC-Cy7 (Clone MI15), and CD27-PE-Cy7 (Clone O321) antibodies were purchased from Biolegend. Anti-human CD8-eFluor 450 (clone OKT8) antibody was obtained from eBioscience.

### Patients and Controls

In total, 34 patients with SLE (diagnosed according to the American College of Rheumatology criteria) (26) and 34 patients with RA (diagnosed according to the American College of Rheumatology and European League Against Rheumatism criteria) (27) who were recruited at the Rheumatology Service of the Hospital Universitario San Vicente Fundación (HUSVF) in Medellín, Colombia were included in this study. Patients with SLE had a median age of 39 (24–54) years, and 85% of these were women. Twenty patients with SLE were classified to have inactive SLE according to the SLE Disease Activity Index (SLEDAI < 4), and 14 patients were classified with active SLE (SLEDAI ≥ 4) (28). The median age of patients with RA was 45 (29–62) years, and 84% of these were women. Sixteen patients with RA were identified to be in remission based on the disease activity score (DAS)−28 (DAS−28 ≤ 2.6); and 18 patients with RA were identified to have moderate activity (DAS−28 > 3.2 and ≤ 5.1). None of the patients received biological therapy. Fourteen patients with SLE and RA each, who had 80% of the disease in the inactive form, were included in our *in vitro* assays with monocyte cells. On the other hand, 10 patients with seropositive RA and 10 with active SLE were included in the MP and MP-IC groups; Additionally, fourteen healthy controls (HC), matched for sex and age, were included. This study was conducted in accordance with the Declaration of Helsinki; the research protocol and informed consent forms were approved by the Universidad de Antioquia's Medical Research Institute and HUSVF Ethic Committees. All patients and HC provided consent for participation in the study.

### MP Isolation and MP-IC Formation

Circulating MP and MP-IC from patients with SLE (LMP and LMP-IC, respectively) and MP and MP-IC from patients with RA (RMP, and RMP-IC, respectively) from poor-platelet plasma were obtained as previously described (4) and were frozen at −70°C until use. Every batch of MP and MP-IC were generated by mixing respective vesicles from 3 to 4 patients. These patients belong to previously published cohorts, in which a detailed characterization of MP was performed. Because the formation of IC by MP was one of the main characteristic associated with the clinical involvement of both SLE (active disease by SLEDAI) (4) and RA (systemic inflammation by inflammatory cytokines) (29) patients in our previous studies, this was the variable specifically evaluated in the present work for MP. The phenotypic characteristic of the MP and MP-IC *ex vivo* before their storage and *in vitro* opsonization are shown in Supplementary Table 1 and Supplementary Figure 1A MP-IC pools were those that formed ≥28.45% of IC for RA patients and ≥38.85% for SLE; MP pools were those that formed ≤ 6% of IC (Supplementary Figure 1B). The MP-IC thresholds were established according to the distribution of the circulating MP-IC frequency in a population of patients with SLE (4) and RA (29); the MP thresholds were established according to the distribution of the circulating MP-IC frequency in a population of HC (4), which was previously studied by us. To MP-IC formation the total IgG was previously obtained from pooled serum samples taken from 16 seropositive patients with SLE [with high levels of antinuclear antibodies (ANAs), anti-DNA and/or anti-Smith] and 16 seropositive patients with RA [with high levels of anti-cyclic citrullinated peptides antibodies (anti-CCP)] by using a NAb™ Protein G Spin Kit (Thermo scientific, Waltham, MA) according to the manufacturer's instructions. IgG enrichment was verified by protein electrophoresis with silver staining and western blot (data not shown). The final IgG preparation of SLE patients used for opsonization had 1:1.280 ANAs [speckled pattern, indirect immunofluorescence (IIF) using HEP-2 cells], 1:40 anti-DNA (IIF), 1220 Units anti-Smith (ELISA), 1270 Units anti-Ro/SSa (ELISA), 90 Units anti-La/SSb (ELISA), and 7630 Units anti-ribonucleoprotein (RNP, ELISA). The final IgG preparation of patients with RA used for opsonization had 286.3 Units anti-CCP (CCP3 IgG ELISA) (30). All these kits were purchased from Inova (San Diego, CA). For opsonization, ~1 × 10^6^ LMP and RMP were mixed and incubated with 15-μg/mL purified IgG (from SLE and RA, respectively) for 60 min at 37°C. Unbound antibodies were washed with 1 mL of DPBS at 17,000 × g for 60 min. The MP from patients with SLE and those from patients with RA forming IC (LMP- and RMP-IC, respectively) was assessed after staining with an *F*(*ab*')2 anti-IgG fragment conjugated with Alexa Fluor-488 for 30 min at 4°C (MP-IgG+ > 28%) using flow cytometry as previously described (Supplementary Figure 1B) (4). For use, each batch was thawed and quantified using flow cytometry as previously reported (4).

### Monolayer Culture of CD14+ Phagocytes and Their Differentiation Into MDM

CD14+ cells were enriched and subsequently differentiated into MDM as previously reported (31). Briefly, peripheral blood mononuclear cells (PBMCs) were isolated using Ficoll density gradient centrifugation from defibrinated venous blood samples from HC and patients with SLE and RA. For monocyte adherence, PBMCs containing 1.2 × 10^5^ CD14+ cells were plated in 96-well plates (Corning Incorporated Life Science, Lowell, MA, USA) using 250-μL RPMI-1640 GlutaMAX with 0.5% inactivated autologous serum-depleted MP (iSA-d) (4) for 4 h at 37°C in 5% CO_2_. Subsequently, wells were washed with pre-warmed DPBS plus 0.5% iSA-d to remove non-adherent cells. The adherent cells obtained were cultured in 250-μL RPMI-1640 supplemented with 10% iSA-d plus 100-μg/ml streptomycin and 100-IU/ml penicillin in the presence or absence of MP (RMP or LMP) and MP-IC (RMP- or LMP-IC) in a ratio of 1:3 (cells:MP) for 6 days at 37°C in 5% CO_2_ to facilitate their differentiation into MDM. After the culturing, supernatants were collected and frozen at −20°C until the level of various cytokines [IL-8, IL-6, IL-10, TNF-α, IL-1β, IL12p70, BAFF (B-cell activating factor) and APRIL (A proliferation-inducing ligand)] was assessed. Morphological changes and the expression of differentiation markers were evaluated in MDM using flow cytometry through specific anti-human antibodies against CD36, HLA-DR, CD16, CD14, and CCR2. Cells were blocked (DPBS plus 1% BSA, 0.01% NaN_3_ and 10% inactivated FBS) and stained for 30 min at 4°C followed by washing twice with washing buffer (DPBS plus 1% BSA and 0.01% NaN_3_). Fluorescence minus one (FMO) controls were established for each antibody using HLA-DR staining. Using the LSR Fortessa flow cytometer with the FACS DIVA software (BD), 50,000 cells were immediately acquired.

In other experiments, the frequency of phagocytic cells, repolarization to M1 and M2 profile, and autologous B- and T- cell co-cultures were performed as detailed below.

### Phagocytosis Assay

To evaluate the phagocytosis of MDM differentiated with or without of MP (RMP or LMP) and MP-IC (RMP- or LMP-IC), MDM were incubated with fluorescent latex beads in a ratio of 1:5 (cell:beads). Subsequently, macrophages were centrifuged for 5 min at 900 × g and incubated for 2 h at 37°C in 5% CO_2_. Then, MDM were repeatedly washed with DPBS and were stained with anti-HLA-DR antibody; ~30,000 cells were immediately acquired using a flow cytometer.

### MDM Repolarization to M1 and M2 Profiles

To evaluate repolarization of MDM differentiated with or without of MP (RMP or LMP) and MP-IC (RMP- or LMP-IC), phagocytes differentiated with and without these extracellular vesicles were treated for 6 h with 20-ng/mL hrIL-4 or 20-ng/mL hrIFN-γ at 37°C in 5% CO_2_ to favor M1 and M2 activation, respectively. Supernatants were collected and frozen at −20°C until the assessment of proinflammatory (M1 markers: IL-8, IL-6, TNF-α, IL-1β, and IL12p70) and anti-inflammatory (M2 marker: IL-10) cytokines. In MDM, M1, and M2 polarization was additionally determined by measuring the expression of classical M1 (CD80, CD86, TLR4, and CD32) and M2 (CD163 and CD209) membrane receptors. MDM staining using specific antibodies was performed as previously detailed. Assays with MDM from HC using different IL-4 and IFN-γ concentrations at differing stimulation times were previously performed to determine culture conditions for M1 and M2 activation. Treatment with 20-ng/mL IFN-γ (M1) or IL-4 (M2) for 6 h at 37°C in 5% CO_2_ was selected (Supplementary Figure 2; Data not shown).

### Co-culture of MDM With Autologous T and B Cells

To evaluate the effect of MDM differentiated with MP (RMP or LMP) and MP-IC (RMP-or LMP-IC) on B- and T-cell activation, fresh autologous CD3+ and CD19+ cells were enriched with the Rosette Sep according to the manufacturer's instructions (purity > 95% and > 90%, respectively). T cells were independently labeled with 1-μM CFSE and repeatedly washed; T and B lymphocytes were co-cultured with previously differentiated MDM with and without extracellular vesicles in RPMI-1640 GlutaMAX that was supplemented with 10% FBS, 100-μg/mL streptomycin and 100-IU/mL penicillin, 10-ng/mL rhIL-2, and 2-mM L-glutamine (complete medium).

For co-culturing with CD3+ lymphocytes, the resultant T cells were left unstimulated (complete medium) or were stimulated with 10-μg/mL PHA and immediately added to pre-washed MDM (2:1, T cells:MDM) and incubated for 96 h at 37°C in 5% CO_2_. Four hours before terminating the incubation of cell cultures, cells were treated with 1-μg/mL Brefeldin A. Lymphocytes were harvested by subjecting them to multiple washes with DPBS and subsequently blocking and staining with anti-CD4 and -CD8 antibodies for 30 min at 4°C. These cells were fixed with 2% paraformaldehyde and permeabilized with 0.5% Tween-20 and 0.2% BSA for 30 min. Subsequently, cells were stained with specific anti-human IFN-γ and TNF-α antibodies for 1 h.

For co-culturing with CD19+ lymphocytes, the resultant B cells were left unstimulated (complete medium) and immediately added to pre-washed MDM cultures (2:1, B cells:MDM) and incubated for 96 h. In parallel, as controls for B-cell activation (Supplementary Figure 3), B cells were unstimulated (complete medium) or stimulated with 2.5-μg/mL affinity-purified F(ab')_2_ fragment anti-human IgM plus 1-μg/mL rhCD40L. Supernatants were collected and frozen at −20°C until the assessment of IgG and IgM levels. Lymphocytes were harvested by multiple washes with DPBS and blocked and stained with anti-human CD19, CD20, CD38, CD27, CD138, CD80, CD86, CD69, and CD95 antibodies for 30 min at 4°C.

Finally, in both cases, 50,000 cells were acquired using a flow cytometer. FMO controls were also included. Cell viability was evaluated using the LIVE-DEAD probe and by changes in FSC-A and SSC-A parameters.

### Cytokine and Immunoglobulin Levels

The Human Inflammatory CBA kit was used to determine the levels of IL-8, IL-6, IL-10, TNF-α, IL-1β, and IL12p70 based on the manufacturer's instructions.

The levels of the cytokines BAFF and APRIL in supernatants were determined using commercial ELISA kits (Quantikine ELISA Human BAFF/BLyS/TNFS13B Kit and DuoSet ELISA Human APRIL/TNFS13 Kit, respectively; R&D Systems) in accordance with the manufacturer's instructions.

IgM and IgG antibody levels in supernatants were determined using commercial ELISA kits (Human IgM Uncoated ELISA kit and Human IgG Uncoated ELISA kit; Invitrogen by Thermo Fisher Scientific, Vienna, Austria) according to the manufacturer's instructions.

### Data Analysis

Two different categorically independent variables were compared using two-way ANOVA (ANOVA II) and the Bonferroni *post-hoc* test (data are presented as mean ± SD). Comparisons among groups of HC and patients with RA and SLE were performed via the Kruskal–Wallis test and Dunn's *post-hoc* test (data are presented as median ± interquartile range). Gating analyses, cell frequencies, mean fluorescence intensity (MFI) and the percentage of divided T cells after proliferation (proliferation modeling algorithm) were estimated using the FlowJo 10.2 software. Statistical analysis was performed using the GraphPad Prism version 7.2 (GraphPad Soft-ware Inc., San Diego, CA, USA). Statistical significance was set at *p* ≤ 0.05; ^*^p ≤ 0.05, ^**^p ≤ 0.01, ^***^p ≤ 0.001, and ^****^p ≤ 0.0001.

## Results

### MP-IC Induce the Differentiation of MDM to a Proinflammatory (M1-Like) Profile

**Figure 1:**
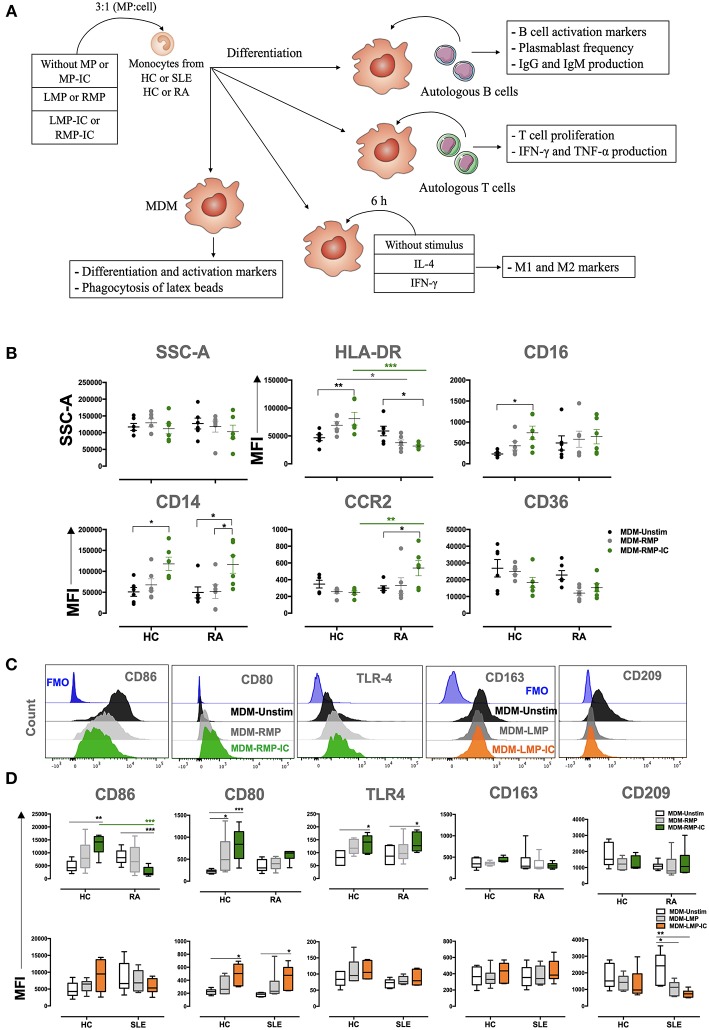
MP-IC change the expression of differentiation and activation markers in MDM. **(A)** Summarized methodological strategy used in this article is presented. **(B)** The MFI of markers associated with the differentiation of MDM: CD36, HLA-DR, CD16, CD16, CCR2, and SSC-A; MDM differentiated without [Unstimulated (Unstim), black dots] or with RMP (light gray dots) or RMP-IC (green dots) from patients with RA (*n* = 6) and HC (*n* = 6). **(C)** Representative histograms of markers associated with M1 polarization (from left to right: CD86, CD80, and TLR4) in MDM from patients with RA differentiated without (Unstim, black histograms) or with RMP (light gray histograms) or RMP-IC (green histograms); representative histograms of markers associated with M2 polarization (CD163 and CD209) in MDM from patients with SLE differentiated without (Unstim, black histograms) or with LMP (dark gray histograms) or LMP-IC (orange histograms). Blue histograms represent the FMO control for each marker. **(D)** Top, the MFI of markers associated with M1 and M2 polarizations in MDM from patients with RA (*n* = 6) and HC (*n* = 6) differentiated without (Unstim, black whisker box) or with RMP (light gray whisker box) or RMP-IC (green whisker box). Below, the MFI of markers associated with M1 and M2 polarizations in MDM from patients with SLE (*n* = 6) and HC (*n* = 6) differentiated without (Unstim, black whisker box) or with LMP (dark gray whisker box) or LMP-IC (orange whisker box). Comparisons among the groups were performed using ANOVA II and the Bonferroni *post-hoc* test.

The methodological strategy of this study is summarized in Figure 1A. The effect of MP and MP-IC in MDM, regarding changes in morphology (side and forward scatter), the expression of differentiation (HLA-DR, CD16, CD14, CCR2, and CD36) and activation (CD86, CD80, TLR-4, CD163, and CD209) markers and the expression of cytokine levels (IL-1β, IL-6, TNF-α, IL-10, IL-8, and IL12p70) were evaluated in mononuclear phagocytes obtained from HC and patients with RA and SLE, differentiated without (MDM-Unstimulated, -Unstim) or with extracellular vesicles (MP from SLE: MDM-LMP and MDM-LMP-IC; MP from RA: MDM-RMP and MDM-RMP-IC). No changes were observed in the granularity (side scatter) and size (forward scatter) of MDM among all the study groups (Figures 1B, Supplementary Figure 4, and data not shown).

**Figure 2:**
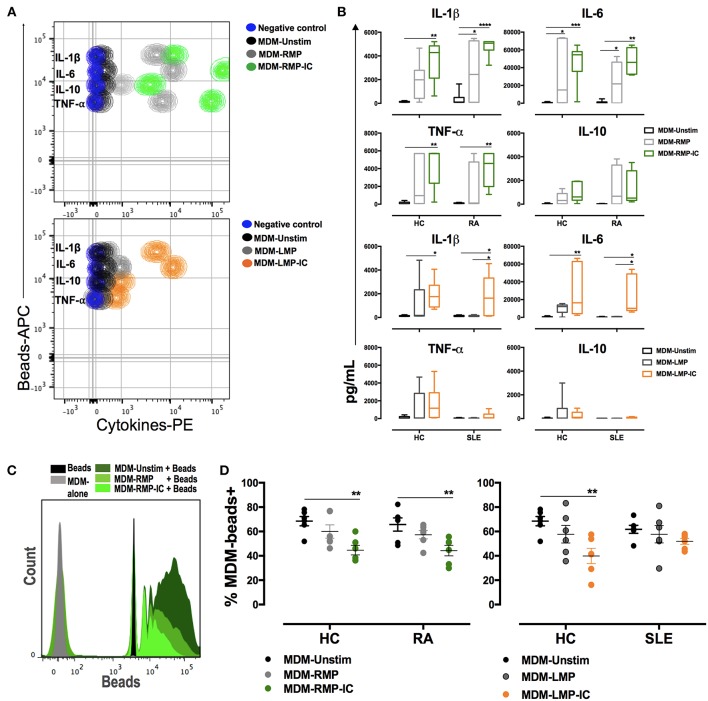
MP and MP-IC induce the polarization of MDM toward a proinflammatory profile compatible with M1 activation and decrease the frequency of macrophages that phagocytose. **(A)** Top, CBA representative contour plot of cytokine levels in the supernatants of MDM from patients with RA differentiated without (Unstim, black contour plot) or with RMP (light gray contour plot) and RMP-IC (green contour plot). Below, a CBA representative contour plot of cytokine levels in the supernatants of MDM from patient with SLE differentiated without (Unstim, black contour plot) or with LMP (dark gray contour plot) and LMP-IC (orange contour plot). In both plots, the negative control (CBA beads alone) is shown as a blue contour plot. **(B)** The first two panels, cytokine levels in the supernatants of MDM from patients with RA (*n* = 6) and HC (*n* = 6) differentiated without (Unstim, black bar graph) or with RMP (light gray bar graph) and RMP-IC (green bar graph). The last two panels, cytokine levels in supernatants of MDM from patients with SLE (*n* = 6) and HC (*n* = 6) that were differentiated without (Unstim, black bar graph) or with LMP (dark gray bar graph) and LMP-IC (orange bar graph). **(C)** Representative histograms of latex beads alone (black); MDM from RA patients differentiated in absence of extracellular vesicles and incubated with fluorescent latex beads (Unstim, dark gray histogram) and MDM differentiated with RMP (medium green histogram) or RMP-IC (light green histogram) and incubated with fluorescent latex beads. **(D)** Left, the frequency of MDM from HC (*n* = 6) and patients with RA (*n* = 6) that bound/internalized fluorescent latex beads after these cells were differentiated without (Unstim, black dots) or with RMP (light gray dots) and RMP-IC (green dots). Right, the frequency of MDM from HC (*n* = 6) and patients with SLE (*n* = 6) that bound/internalized fluorescent latex beads after these cells were differentiated without (Unstim, black dots) or with LMP (dark gray dots) or LMP-IC (orange dots). Comparisons among the groups were performed using ANOVA II and the Bonferroni *post-hoc* test.

MDM from HC showed increased HLA-DR, CD14, CD16, CD86, CD80, and TLR-4 expression in addition to increased IL-1β, IL-6, and TNF-α levels when these cells were differentiated with RMP-IC compared with those differenced with MDM-Unstim cells (Figures 1B–D, 2B). Only CD16 and CD80 expressions and IL-1β and IL-6 levels increased in MDM from HC that were exposed to LMP-IC (Figures 1D, 2A,B and Supplementary Figure 4). These results showed that MP-IC promote the differentiation of MDM from HC to a proinflammatory (M1-like) profile; this phenomenon was more evident with the vesicles of patients with RA than SLE.

With respect to mononuclear phagocytes from patients with RA, MDM differentiated with RMP-IC increased CD14, CCR2, and TLR4 expressions as well as IL-1β, IL-6, and TNF-α levels in supernatants but decreased HLA-DR and CD86 expressions compared with those differentiated with MDM-Unstim cells (Figures 1B–D, 2A,B). Conversely, increased CD80 expression and decreased CD209 expression and high IL-1β and -6 levels in supernatants were noted in the MDM of patients with SLE that were differentiated with LMP-IC compared with those that were differentiated with MDM-Unstim cells (Figures 1C,D, 2A,B and Supplementary Figure 4). These results suggested that MP-IC also promote the proinflammatory differentiation of mononuclear phagocytes in RA and SLE patients.

As an indirect measure of macrophage M1 or M2 activation (17), the frequency of MDM that bind/internalize latex beads was evaluated after their differentiation with or without MP and MP-IC. Few positive cells were found to be bound to latex beads when MDM from HC were exposed to RMP-IC and LMP-IC (Figures 2C,D). Similar results were observed with MDM from patients with RA, but no changes were noted in the cells of patients with SLE (Figures 2C,D). These results corroborated that the presence of MP-IC allow the differentiation of MDM to a M1-like profile. These changes were more evident in MDM from HC than in those from patients with RA and SLE.

### MDM Differentiated With RMP-IC Were Resistant to Repolarization to M2-Like Profiles Following IL-4 Treatment

**Figure 3:**
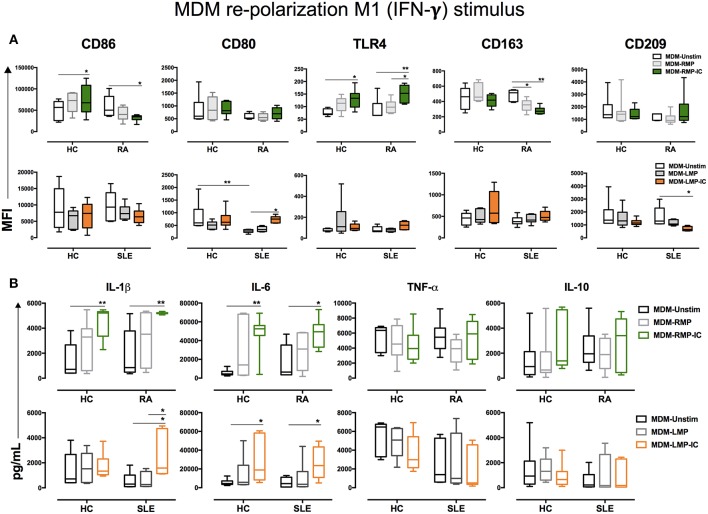
IFN-γ enhances the proinflammatory profile of MDM differentiated with MP. **(A)** Top, the MFI of markers associated with M1 and M2 polarizations in MDM from patients with RA (*n* = 6) and HC (*n* = 6) that were differentiated without (Unstim, black whisker box) or with RMP (light gray whisker box) and RMP-IC (green whisker box) along with 6 h of IFN-γ treatment. Below, the MFI of markers associated with M1 and M2 polarizations in MDM from patients with SLE (*n* = 6) and HC (*n* = 6) differentiated without (Unstim, black whisker box) or with LMP (dark gray whisker box) and LMP-IC (orange whisker box) along with 6 h of IFN-γ treatment. **(B)** Top, cytokine levels in the supernatants of MDM from patients with RA (*n* = 6) and HC (*n* = 6) differentiated without (Unstim, black bar graph) or with RMP (light gray bar graph) and RMP-IC (green bar graph) along with 6 h of IFN-γ treatment. Below, cytokine levels in supernatants of MDM from patients with SLE (*n* = 6) and HC (*n* = 6) differentiated without (Unstim, black bar graph) or with LMP (dark gray bar graph) and LMP-IC (orange bar graph) along with 6 h of IFN-γ treatment. Comparisons among the study groups were performed using ANOVA II and the Bonferroni *post-hoc* test.

**Figure 4:**
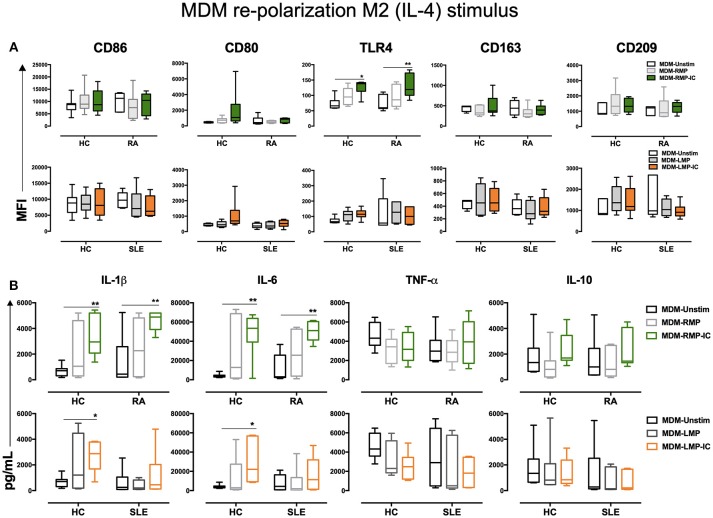
IL-4 does not re-polarize the proinflammatory profile of MDM from HC and patients with RA differentiated with MP and MP-IC. **(A)** Top, the MFI of markers associated with M1 and M2 polarizations in MDM from patients with RA (*n* = 6) and HC (*n* = 6) differentiated without (Unstim, black whisker box) or with RMP (light gray whisker box) and RMP-IC (green whisker box) along with 6 h of IL-4 treatment. Below, the MFI of markers associated with M1 and M2 polarizations in MDM from patients with SLE (*n* = 6) and HC (*n* = 6) differentiated without (Unstim, black whisker box) or with LMP (dark gray whisker box) and LMP-IC (orange whisker box) along with 6 h of IL-4 treatment. **(B)** Top, cytokine levels in supernatants of MDM from patients with RA (*n* = 6) and HC (*n* = 6) differentiated without (Unstim, black bar graph) or with RMP (light gray bar graph) and RMP-IC (green bar graph) along with 6 h of IL-4 treatment. Below, cytokine levels in supernatants of MDM from patients with SLE (*n* = 6) and HC (*n* = 6) differentiated without (Unstim, black bar graph) or with LMP (dark gray bar graph) and LMP-IC (orange bar graph) along with 6 h of IL-4 treatment. Comparisons among the groups were performed using ANOVA II and the Bonferroni *post-hoc* test.

To determine whether MDM differentiated with MP and MP-IC could reverse or potentiate their proinflammatory profiles, these MDM were further treated with IFN-γ or IL-4. As expected, IFN-γ treatment enhanced the MFI of M1-related molecules, such as CD86 and CD80, as well as TNF-α levels compared with those without treatment (Data not shown). However, IFN-γ treatment had no additive effect on MDM differentiated with MP and MP-IC regarding CD86, CD80, and TLR-4 expressions and IL-1β, IL-6, TNF-α, and IL-10 production (Figures 3A,B). IL-4 treatment did not result in an increase in CD86 and CD80 expressions in MDM of HC differentiated with RMP-IC, as noted in previous results (Figure 1C). However, after IL-4 treatment the TLR-4 expression and IL-1β and IL-6 levels remain increased in MDM of HC and patients with RA differentiated with RMP-IC. No differences were noted regarding TNF-α and IL-10 levels (Figures 4A,B). In patients with SLE, when MDM were differentiated with LMP-IC, IL-1β, IL-6, and TNF-α levels did not increase after additional IL-4 treatment (Figures 4A,B), suggesting that these cells, but not MDM from HC and patients with RA, reversed the M1 profile induced by MP-IC (Supplementary Figure 5). These negligible changes cannot be explained due to a low effect of IL-4, since we observed that MDM treated only with IL-4 up regulated CD163, CD209, and IL-10, a phenotype compatible to M2 activation (Supplementary Figure 2).

The aforementioned results showed that RMP-IC induce a more prominent differentiation to an M1-like profile than LMP-IC in the MDM of HC and patients with RA (Supplementary Figure 4).

### MDM Differentiated With LMP-IC and RMP Induce Further Proliferation of Activated CD4+ T Cells From Patients With SLE and RA, Respectively

**Figure 5:**
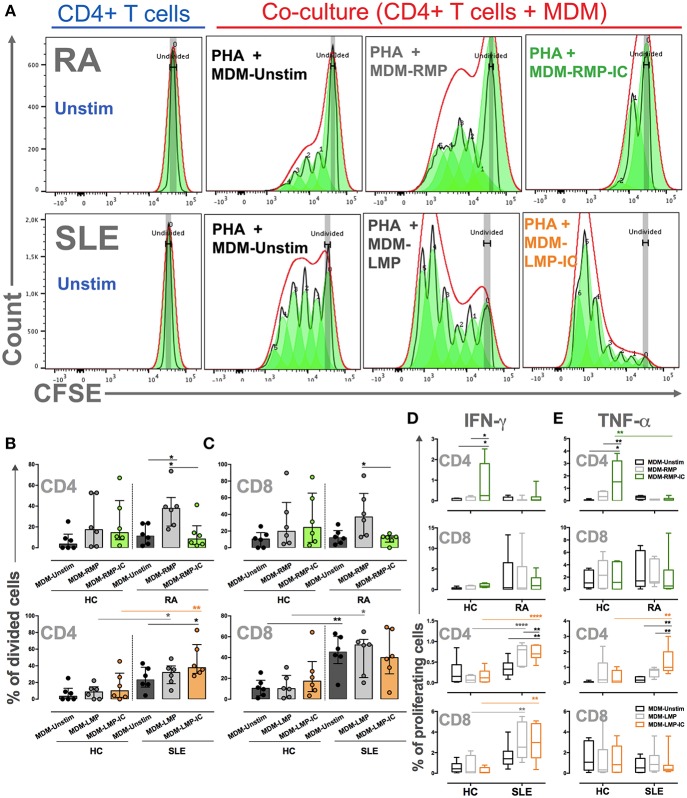
MDM differentiated with MP and MP-IC induce the proliferation and activation of autologous T cells. **(A)** Representative proliferation modeling of CD4+ T cells alone (in complete medium) without (Unstim) or with stimulus with PHA and co-cultured with MDM from patients with RA (Top panel) and SLE (Below panel) differentiated without (MDM-Unstim) or with MP and MP-IC. **(B,C)** The frequency of CD4+ T cells **(B)** and CD8+ T cells **(C)** from patients with RA and SLE and HC that divided after PHA treatment and co-culture with MDM differentiated without (Unstim) or with MP or MP-IC. **(D)** The frequency of proliferating IFN-γ+ CD4+ T cells and IFN-γ+ CD8+ T cells and **(E)** the frequency of proliferating TNF-α+ CD4+ T cells and TNF-α+ CD8+ T cells from patients with RA and SLE and HC after PHA treatment and co-culture with MDM differentiated without (Unstim) or with MP and MP-IC. In all cases, patients with SLE: *n* = 6, patients with RA: *n* = 6, and HC: *n* = 6; comparisons among the groups were performed using ANOVA II and the Bonferroni *post-hoc* test.

To evaluate the effects of MDM differentiated in the presence of MP and MP-IC on T-cell activation, MDM differentiated with or without these vesicles were co-cultured with T cells and stimulated with PHA. In patients with RA, MDM differentiated with RMP induced a higher proportion of dividing CD4+ T cells compared with those differentiated without extracellular vesicles or RMP-IC (Figures 5A,B). The induction of IFN-γ+ and TNF-α+ CD4+ T cells was not noted, and cytokine responses of CD8+ T cells to MDM from patients with RA differentiated with RMP and RMP-IC were not observed (Figures 5B–E). Additionally, no differences were detected in the frequency of live T cells in co-cultures (≥80%) compared with cells without MDM (≥83%). Notably, in MDM from HC differentiated with RMP-IC, an increased frequency of CD4+ T cells producing IFN-γ and TNF-α were observed.

MDM from patients with SLE differentiated with LMP-IC demonstrated a higher percentage of proliferating CD4+ T cells and an increased frequency of IFN-γ+ and TNF-α+ in CD4+ proliferating T cells than those in MDM differentiated without extracellular vesicles (Figure 5). Interestingly, although CD8+ T cells from patients with SLE proliferated in response to MDM regardless of the presence of LMP and LPM-IC, an increased percentage of IFN-γ+ cells were observed in these lymphocytes when MDM were differentiated with LMP and LPM-IC compared with those differentiated without these vesicles (Figure 5). Interestingly, T cells from HC did not respond to either MDM differentiated with or without these extracellular vesicles from SLE patients.

These results showed that MDM differentiated with extracellular vesicles favor CD4+ and CD8+ T-cell activation of patients with autoimmune diseases.

### MDM Differentiated With MP-IC Induce the Activation and Survival of B Cells From Autoimmune Patients

**Figure 6:**
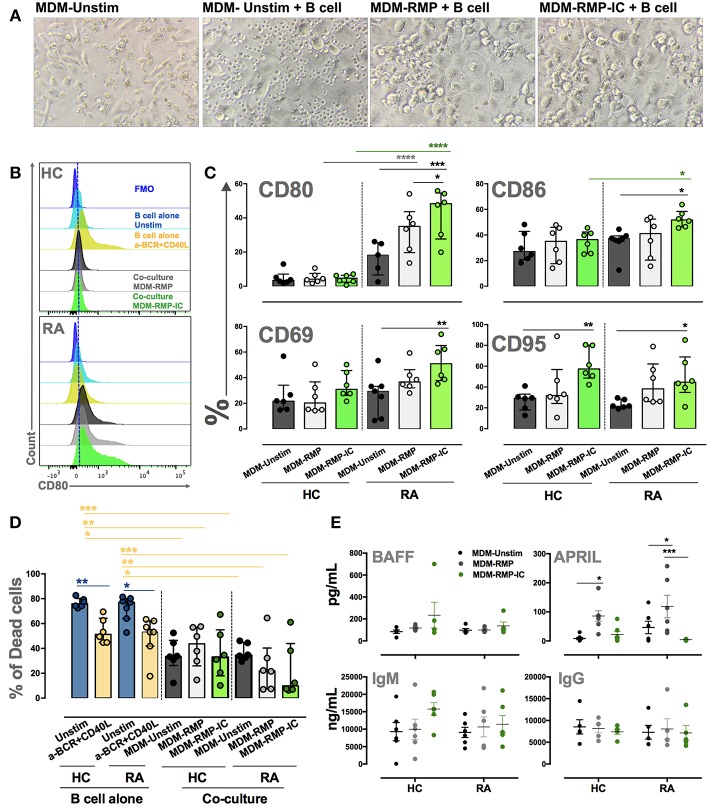
MDM differentiated with MP-IC, and mainly with MP, induce the activation of autologous B cells from patients with RA. **(A)** From left to right: representative light microscopy pictures of MDM unstim alone; MDM unstim co-cultured with B cells; MDM differentiated in the presence of RMP or RMP-IC from patients with RA and co-cultured with B cells. **(B)** Representative histograms of CD80 expression on B cells from HC (top) and patients with RA (below) cultured alone (light blue) and with anti-BCR plus CD40L (yellow) or co-cultured with MDM differentiated without (Unstim, black) or with RMP (gray) and RMP-IC (green). Blue histograms represent the FMO control. **(C)** The frequency of CD80, CD86, CD69, and CD95 in B cells from patients with RA (*n* = 7) and HC (*n* = 6) co-cultured with MDM differentiated without (Unstim) or with RMP and RMP-IC. **(D)** The frequency of dead B cells (positive for LIVE-DEAD probe) from patients with RA (*n* = 7) and HC (*n* = 6) cultured alone (Unstim, in complete medium) and with anti-BCR plus CD40L (positive control) or co-cultured with MDM differentiated without (Unstim) or with RMP and RMP-IC. **(E)** BAFF and APRIL (Top panel) levels in supernatants of MDM from patients with RA (*n* = 5) and HC (*n* = 5) differentiated without (Unstim) or with RMP and RMP-IC. IgG and IgM (below panel) levels in supernatants from co-cultures of MDM differentiated with or without RMP and RMP-IC with autologous B cells from HC (*n* = 5) and RA (*n* = 5) patients. Comparisons among the groups were performed using ANOVA II and the Bonferroni *post-hoc* test.

The co-culture of MDM differentiated with RMP-IC and B cells from patients with RA increased the expression of activation markers CD80, CD86, CD69, and CD95 on these lymphocytes compared with the co-culture of MDM differentiated without vesicles and B cells from patients with RA (Figures 6A–C). This activation phenotype was associated with a significant decrease in the frequency of dead B cells in co-cultures but not with an increase in BAFF levels in the supernatants of MDM from patients with RA differentiated with RMP-IC (Figures 6D,E). In addition, no differences were noted in the frequency of plasmablasts and plasmatic cells (Supplementary Figure 3B and data not shown) or the induction of IgM and IgG production by B cells from patients with RA in the co-cultures (Figure 6E). Interestingly, MDM differentiated with RMP, and not with RMP-IC, increased APRIL levels (Figure 6E). Moreover, MDM from HC differentiated with RMP-IC only increased CD95 level in B cells and induced IgM secretion. These cells were also more prone to cell death and showed no change in BAFF, APRIL, and IgG level compared with those from patients with RA (Figure 6).

**Figure 7:**
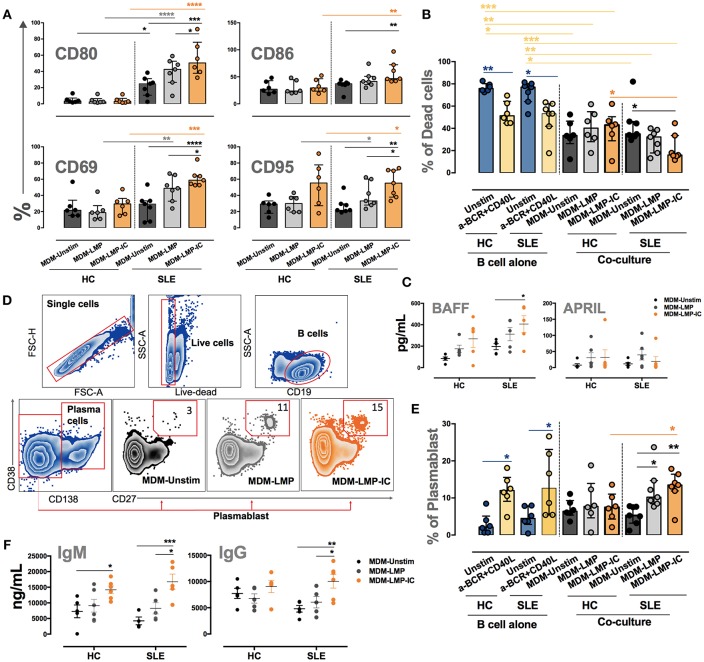
MDM differentiated with MP and MP-IC induce the activation and plasmablast differentiation of autologous LB from patients with SLE. **(A)** The frequency of CD80, CD86, CD69, and CD95 in B cells from patients with SLE (*n* = 7) and HC (*n* = 6) co-cultured with MDM differentiated without (Unstim) or with LMP and LMP-IC. **(B)** The frequency of dead B cells (positive for LIVE-DEAD probe) from patients with SLE (*n* = 7) and HC (*n* = 6) cultured alone (Unstim, in complete medium) and with anti-BCR plus CD40L (positive control) or co-cultured with MDM differentiated without (Unstim) or with LMP and LMP-IC. **(C)** BAFF and APRIL levels in the supernatants of MDM from patients with SLE (*n* = 5) and HC (*n* = 5) differentiated without (Unstim) or with LMP and LMP-IC. **(D)** Representative gating strategy to determine the frequency of plasmablasts after the co-culture of B cells with MDM differentiated without (Unstim) or with LMP and LMP-IC. **(E)** The frequency of plasmablasts from B cells cultured alone (Unstim, in complete medium), with anti-BCR plus CD40L (positive control), or co-cultured with autologous MDM from patients with SLE (*n* = 7) and HC (*n* = 6) differentiated without (Unstim) or with LMP and LMP-IC. **(F)** IgG and IgM levels in supernatants from the co-cultures of MDM differentiated with or without LMP and LMP-IC with autologous B cells from HC (*n* = 5) and patients with SLE (*n* = 5). Comparisons among the groups were performed using ANOVA II and the Bonferroni *post-hoc* test.

The co-culture of B cells from patients with SLE and MDM differentiated with LMP-IC increased the expression of activation markers CD80, CD86, CD69, and CD95 on these lymphocytes compared with the co-culture of MDM differentiated without vesicles and B cells from patients with SLE (Figure 7A). This activation phenotype was associated with a significant decrease in the frequency of dead B cells and with an increase in BAFF levels in the supernatants of MDM from patients with SLE differentiated with LMP-IC (Figures 7B,C). In addition, all this was related to an increased plasmablast frequency, but not to plasmatic cells, and high IgM and IgG levels in co-cultures of B cells of patients with SLE and MDM differentiated with LMP-IC (Figures 7D–F; data not shown). MDM from HC differentiated with LMP or LMP-IC tended to exhibit increased BAFF levels; moreover, these cells induced increased IgM secretion without a significant trend in the increase of CD95 level in B cells.

These results demonstrated that MDM differentiated in the presence of MP-IC can induce activation and survival of B cells of patients with autoimmune diseases.

## Discussion

Macrophages are crucial in the pathogenesis of RA and SLE (16, 32). In the context of an inflammatory response, tissues recruiting blood monocytes are considered as the sources of inflammatory macrophages (10, 33), and M1- and M2-like disequilibrium of macrophages results in chronic inflammation (16, 34). In SLE (4, 7) and RA (29), MP and MP-IC exert proinflammatory effects in monocytes and MDM. However, to the best of our knowledge, this is the first report about the effect of MP and MP-IC on mononuclear phagocytes differentiation. Therefore, the findings of this study support the idea that the uptake of MP-IC in patients with RA and SLE by monocytes biases the differentiation toward M1-like MDM along with the expansion of proinflammatory responses and the induction of lymphocyte activation. Then, the effect of MP-IC in M1-like differentiation of MDM may contribute to the chronic inflammatory process and promote adaptive responses in systemic autoimmune diseases.

### MP-IC Induce the Differentiation of Monocytes Into M1-Like MDM

Our results agreed with previous reports indicating proinflammatory effects of MP in different immune system cells (5, 35, 36). Despite a number of studies have shown that macrophages from patients with SLE have intrinsic defects in the phagocytosis of latex beads, apoptotic cells, bacteria, and yeast (31, 37, 38); the phagocytosis of IC is intact or even increased in these patients compared with that in patients with non-opsonized antigens (4, 39). In our previous studies, we demonstrated a more efficient uptake of MP-IC than that of MP by monocytes from patients with SLE (4) and RA (29); this may explain the higher proinflammatory response observed in MDM differentiated with MP-IC than those differentiated with MP. In addition, MP-IC induce the production of proinflammatory cytokines IL-6, IL-1β, TNF-α (RA and SLE), and IFN-α (SLE) in mononuclear phagocytes from patients with RA (29) and SLE (4). Considering these findings and that MP-IC induced higher proinflammatory differentiation than MP, it can be expected that signaling mechanisms and processing of these vesicles are similar to those reported for IC, for example the cross-linking of FcγR (CD16, CD32, and CD64) (12, 40).

**Figure 8:**
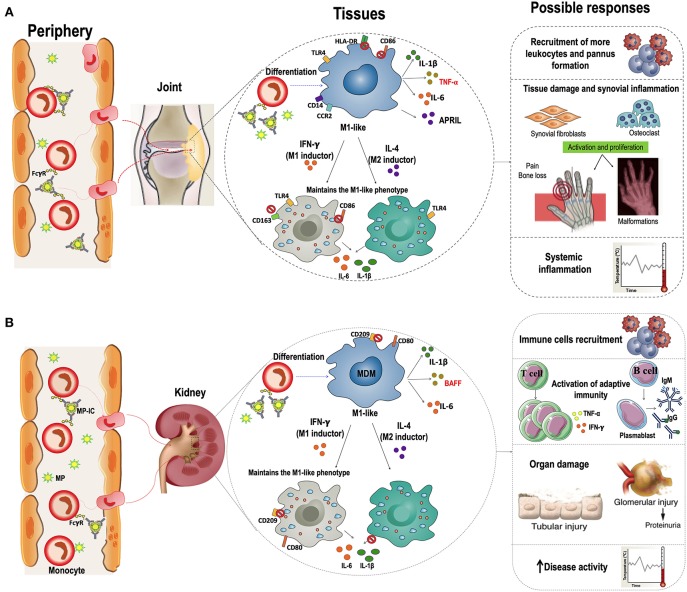
Hypothetical models of the effect of macrophages differentiated in the presence of MP-IC in the RA and SLE pathogenesis. Proposed hypothetical model of the dynamic of interaction between MP-IC and monocytes in circulation and their potential contribution to promote macrophages differentiation to a proinflammatory profile in the tissues of patients with seropositive RA **(A)** and patients with active SLE **(B)**; these activated macrophages contribute to T cell activation and expansion, and B cell survival and production of antibodies; therefore, MP and specially MP-IC can participate in the pathophysiology of these autoimmune diseases, favoring the perpetuation of the chronic inflammation and autoimmune responses that affect the tissues of RA (joint, **A**) and SLE (kidney, **B**) patients.

Interestingly, IC can also induce monocyte differentiation; Tanaka et al. described that monocytes from healthy individuals differentiated into an immature dendritic cells (iDCs)-like phenotype in the presence of plate-immobilized human IgG (as a model for IC), and produce several proinflammatory cytokines, such as TNF-α, IL6, and GM-CSF, through the activation of FcγRI (CD64). These cells trigger autologous T-cell proliferation and cytokine production, including IFN-γ, TNF-α, and IL-4 (41). Several studies have shown that IC containing IgG against citrullinated peptides (ACPA-IC) induce FcγRIIa (CD32)-mediated TNF-α secretion in macrophages from patients with RA (42–45). Furthermore, in *in vitro* and *in vivo* models of SLE pathogenesis, IC can bind to FcγRs expressed on the surface of monocytes and plasmacitoid (p)-DCs. Their subsequent internalization allowed the DNA present in these IC to activate TLR9, inducing the production of proinflammatory cytokines, mainly TNF-α, IL-10, and IFN-α (46, 47). These studies support the hypothesis that MP-IC can activate mononuclear phagocytes. In addition, our results not only agree with these reports but also showed the effect of MP-IC in the proinflammatory differentiation of MDM. Therefore, elevated amounts of circulating MP-IC in patients with active SLE and seropositive RA might have an impact in the proinflammatory differentiation of monocytes in patients once these cells migrate from blood to different tissues (Figure 8).

Although the proinflammatory differentiation of MDM was higher with MP-IC than that with MP, differences were also noted according to the source of monocytes. There were more prominent responses with monocytes from HC than that with monocytes from patients with RA and SLE. FcγR expression on circulating monocytes in patients with RA and SLE differed from those in HC. Several reports have shown an increased expression of CD64 in monocytes from patients with SLE (4, 48, 49), whereas CD32 expression was more predominant in monocytes from patients with RA (50). In addition, an imbalance was noted in the circulating monocyte subsets of these two diseases, both in murine models and in patient samples. Reportedly, intermediate monocyte subset in RA and non-classical subset in SLE seem to primarily migrate toward inflammation sites (9, 51). These monocytes are quite heterogeneous with regard to their phenotype and function and can respond in different ways to the same stimulus (10). These differences in both the monocyte phenotypes and sources may partly explain the variations observed in the differentiation of MDM from HC and patients with RA and SLE with MP-IC in our system. However, these differences could be also explained by other causes such as trained innate immunity phenomena (52) or immunosuppressive treatment. Thus, more studies are needed to further clarify these differences.

Moreover, although higher proinflammatory cytokine levels were noted during the differentiation of MDM with MP-IC than those with MP, differences were also noted according to the source of these vesicles as mainly appreciated in our HC data. Previously, MP from patients with autoimmune diseases were reported to contain alarmins and other TLRs ligands, such as HMGB1, citrullinated peptides (CPs), nucleic acids, and chromatin (4, 5, 35). In our case, the proinflammatory responses were observed primarily with MP from patients with RA. The content of post-translational modifications, e.g., CPs in RMP (5), can directly activate these phagocytes. CPs stimulate TNF-α, IL-6, IL-1β and IL-8 production by monocytes isolated from patients with RA, and these proinflammatory cytokine levels were abrogated by a TLR4 blockade (53). Therefore, we propose that MP from patients with RA may contain more alarmins, which activate mononuclear phagocytes and promote proinflammatory responses, than those from patients with SLE.

*In vitro* studies have demonstrated that human macrophages polarized to the M1-like phenotype can switch to M2-like phenotype following changes in micro-environmental conditions; this response has been associated with the regulation of inflammation by the production of anti-inflammatory cytokines such as IL-10 and transforming growth factor- (TGF)-β (27). The reversibility of polarization plays a critical physiological and therapeutic role, especially in diseases in which an M1/M2 imbalance plays a pathogenic role, such as RA and SLE. Strikingly, in our *in vitro* model, we found that the MDM of patients with RA and HC differentiated with RMP-IC were refractory to an M2 stimulus (IL-4). Although, IC have been reported to favor differentiation toward an alternative activation of macrophages (M2b) (54), Vogelpoel et al. reported a synergistic upregulation of proinflammatory cytokines TNF-α, IL-1β and IL-6 in macrophages derived from patients with RA and HC by IC exhibiting TLR ligands (55). It is tempting to propose that in chronic autoimmune diseases, such as RA and SLE, in which high levels of MP-IC are present in the circulation, these vesicles may favor a constant differentiation of monocytes into M1-like MDM in inflamed tissues (Figure 8).

### MDM Differentiated With MP and MP-IC Promote T- and B-Cell Activation

One of the most important implications of macrophages in the progression of autoimmune diseases is their function as antigen presenting cells (APCs). Activated monocytes and macrophages from the synovial fluid of patients with RA function as APCs to promote pathogenic CD4 T-cell responses at this inflammation site (56). Furthermore, the correlation of macrophage infiltration and kidney dysfunction in humans supports the contribution of macrophages in SLE (57, 58). Macrophages can perform canonical and non-canonical presentations to T (54) and B cells, respectively (59). Macrophages are considered as major APCs in tissues in second antigenic challenges, without the requirement of recirculation to lymph nodes (60). Considering our results, there may be migrant monocytes in patients with RA and SLE that differentiate into MDM with a M1-like profile in the presence of MP or MP-IC, presenting self-antigens contained in these vesicles to T and B cells localized in target organs (Figure 8).

MP-IC seems to have important implications regarding how monocytes/macrophages modulate the response of adaptive immune cells. Importantly, although extracellular vesicles from patients with RA induced a higher proinflammatory response compared with those from patients with SLE, M1-like macrophages differentiated with RMP and RMP-IC induced a more discrete activation of T and B cells than MDM differentiated with LMP and LMP-IC. This phenomenon can have different explanations such as differences in the treatment regimen and in the amount of autoreactive and memory cells in the evaluated patients (2, 3); it may be associated also with differences in the activation profile, differentiation, epigenetically modifications or other intrinsic defects of these cells in each disease. Therefore, we considered this aspect a limitation of our study. However, these results showed some contrasting manifestations, as previously demonstrated between these patients, such as a more significant involvement of proinflammatory cytokines including TNFα in patients with RA (2, 61), and B cells and antibody producing cells in patients with SLE (3). We found that RMP-IC induced a decreased expression of HLA-DR and CD86 in MDM from patients with RA compared with MDM differentiated without vesicles. This may explain the low CD4+ and CD8+ T-cell activation and proliferation in co-cultures with MDM from patients with RA differentiated with MP-IC compared with those from patients with SLE. Furthermore, it is possible that MDM from patients with SLE make a more efficient semi-direct presentation of these vesicles than those from patients with RA; this antigenic presentation route has been primarily described as one of the mechanisms that govern the rejection of transplants (62) but until now has not been characterized in autoimmune diseases. Thus, the study of new underlying mechanisms and routes of crosstalk between these cells of innate and adaptive immunity is essential to further understand autoimmune diseases and in the design of alternative therapeutic strategies.

Interestingly in RA, only MP but not MP-IC, enhanced T-cell proliferation and activation. Indeed, APRIL was detected only in the supernatants of MDM differentiated with RMP. The APRIL receptor, a transmembrane activator and calcium-modulating cyclophilin ligand interactor (TACI), is expressed on resting and activated mature B cells (63) and activated T cells (64). Wang et al. showed that APRIL neutralization with soluble TACI immunoadhesin (TACI-Fc) *in vitro* inhibits antigen-specific T-cell activation (65). Another possible explanation of our results is related to TLR ligands present in MP (4, 35, 36). Hardenberg et al. showed that human monocyte-derived DCs secrete APRIL but not BAFF using TLR9 and TLR3 ligands (66). A different study demonstrated that synovial fibroblasts from patients with RA produce APRIL in response to TLR3 and TLR4 ligands present in the synovial fluid of these patients (67). Thus, MP-IC, at least in RA, may have contradictory roles in MDM differentiation, including the induction of an M1-like profile rather than T cells responses. RMP alone could expose more CP and induce the production of APRIL via TLR4, whereas in vesicles containing anti-CCP antibodies, there may exist some allosteric impediment for recognition by surface TLR4. However, additional studies are required to support this theory.

We found that the frequency of dead B cells decreases when they are co-cultured with autologous MDM from patients with SLE and RA and HC differentiated with or without MP and MP-IC. Strikingly, higher BAFF levels were detected in the supernatants of MDM from patients with SLE. Reportedly, BAFF is essential for B-cell maturation and survival (68). Inflammatory stimuli, such as IC from patients with SLE, LPS, and IFN-α induce BAFF production by human monocytes and macrophages (69, 70); in addition, monocytes from patients with systemic autoimmune diseases produce more BAFF than monocytes from HC (71). This evidence suggested a potential survival mechanism for autoreactive B cells in patients with SLE, induced by MP and primarily by MP-IC indirectly. However, we should point out that autologous MDM-Unstim cells also promoted the expression of some activation markers and decreased the frequency of dead B cells of patients with RA and SLE, although the effect of MDM differentiated with MP-IC seem to be more significant. Possibly, MDM can provide soluble survival factors besides BAFF, such as IL-15 and TNF-α (72). In addition, the release of other factors such as IL-6 and IL-10 observed in these cultures may also play an important role in B-cell activation and survival.

Plasmablast differentiation was observed in the co-cultures of B cells and MDM from patients with SLE differentiated with LMP and LMP-IC. Previously, Kwissa et al. showed that CD14+CD16+ monocytes infected with dengue virus and their co-cultures with autologous B cells stimulated the differentiation of B cells into CD27^++^CD38^++^ plasmablasts, in a BAFF, TACI, and IL-10 depend manner (73). Other studies have shown that DC and monocytes stimulate B-cell differentiation into plasmablasts with BAFF- and APRIL-dependent mechanisms (74). Although we did not perform blocking assays for these cytokines, we observed that MDM-LMP-IC produce higher concentrations of BAFF in patients with SLE. Thus, this cytokine may be the potential mechanism by which MP and MP-IC indirectly stimulate plasmablast formation in patients with SLE.

Other possible routes via which MDM activate B cells include the non-canonical presentation of antigens and IC to B cells by macrophages (75). In addition, the ability to degrade the internalized cargo is impaired in the monocytes of patients with SLE (76) and in the macrophages of a lupus murine model (77). Monteith et al. showed that macrophages from lupus-prone MRL/*lpr* mice exhibit defective degradation of FcγR-bound cargo, induced by impaired lysosomal maturation and attenuated lysosomal acidification (77). Strikingly, the undegraded material of IC was recycled back to the cell membrane, and macrophages accumulated high levels of these IC on their surface. Considering this, we propose that macrophages from SLE in our model can activate B cells through a non-canonical presentation of extracellular vesicles. These defects have not been reported in phagocytic cells from patients with RA, and future studies are required to support this hypothesis.

## Conclusion

Despite the variability of the cell and MP sources in this study is quite large and increase the variability of results and limiting interpretation of those, we can conclude that MDM differentiated with MP-IC are more prone to an M1-like profile. As a consequence, mononuclear phagocytes differentiated with these vesicles are able to induce T-cell activation and support B-cell survival (hypothetical models presented in Figure 8). Excessive extracellular vesicles in the blood of patients with systemic autoimmune diseases may maintain the expansion of autoreactive T-cell clones and favor the survival of autoreactive B cells. The effect of MP and MP-IC on monocytes and macrophages seems to have clear consequences on the function and response of the adaptive immune system and therefore in the pathophysiology of autoimmune diseases. Our results also reaffirmed the notion that MP are promising therapeutic targets for patients with systemic autoimmune diseases.

## Ethics Statement

This study was conducted in accordance with the Declaration of Helsinki; the research protocol and informed consent forms were approved by the Universidad de Antioquia's Medical Research Institute and HUSVF Ethics Committees. All patients and HCs provided consent for participation in the study.

## Author Contributions

CB and DC contributed to the study design, data acquisition, analysis and interpretation, and manuscript drafting. JV-V performed data acquisition and critical manuscript revision. CM-V performed clinical data acquisition and interpretation. MR and GV performed data analysis and interpretation and critical manuscript revision. All authors approved the final version of the manuscript and agreed to be accountable for all aspects of the work.

### Conflict of Interest Statement

The authors declare that the research was conducted in the absence of any commercial or financial relationships that could be construed as a potential conflict of interest.
