# Supplementary material for: Proinflammatory Differentiation of Macrophages Through Microparticles That Form Immune Complexes Leads to T- and B-Cell Activation in Systemic Autoimmune Diseases
Source: Front Immunol. 2019 Aug 28;10:2058. doi: 10.3389/fimmu.2019.02058 (PMC6724570; doi:10.3389/fimmu.2019.02058)
Supplement: Supplementary file 1 [file Image_1.pdf]

Supplementary Information

Proinflammatory differentiation of macrophages through microparticles that form immune complexes leads to T- and B-cell activation in systemic autoimmune diseases

Catalina Burbano[1,2], Juan Villar-Vesga[1], Gloria Vásquez[1], Carlos Muñoz-Vahos[3], Mauricio Rojas[1,2], Diana Castaño[1*]

[1] Grupo de Inmunología Celular e Inmunogenética, Instituto de Investigaciones Médicas, Facultad de Medicina, Universidad de Antioquia UdeA, Medellín, Colombia

[2] Unidad de Citometría de Flujo, Sede de Investigación Universitaria, Universidad de Antioquia UdeA, Medellín, Colombia

[3]Sección de Reumatología. Hospital Universitario de San Vicente Fundación. Medellín, Colombia

[*]**Correspondence:** DC diana.castano@udea.edu.co

Supplementary Table 1. Phenotypic characteristics of MP from patients with RA and SLE.

	RA		SLE	
**Characteristic***	**RMP**	**RMP-IC**	**LMP**	**LMP-IC**
Count (MP/mL)	**711 136** (435 816 - 9 542 616)	**1 101 600** (220 600 - 6 804 485)	**1 049 375** (294 675 - 9 002 690)	**1 284 875** (213 420 - 7 674 235)
% of CD41a+ (platelets marker)	**62.55** (34.6 - 87.3)	**45.9** (13.7 - 94)	**19.9** (16-5 - 60.2)	**44.05** (13.3 - 74.6)
% of CD45+ (Leukocyte marker)	**40.65** (17.4 - 64.6)	**35.2** (5.08 - 62.1)	**21.7** (19.1 - 29.2)	**34.5** (13.1 - 68.7)
% of CD105+ (Endothelial marker)	**3.35** (1.48 - 18.2)	**4.66** (0.36 - 26.6)	**9.15** (4.01 - 14.3)	**5.14** (1.64 - 23.6)
% of CD235a+ (Erythrocyte marker)	**7.03** (4.96 - 8.94)	**7.91** (0.58 - 19.5)	**7.08** (6.77 - 7.4)	**8.08** (1.57 - 21.6)
% of HLA-DR+	**43.6** (19 - 65.2)	**39.1** (0.74 - 77.9)	**28.7** (16.4 - 38.6)	**39.1** (9.24 - 73.8)
% of Annexin V+	**43.2** (16.6 - 54.5)	**42.4** (9.1 - 80.6)	**36.6** (11.4 - 56.6)	**34.4** (2.26 - 91.8)
% of DNA+	**2.8** (0.5 - 6)	**1.6** (0 - 24.3)	**0.5** (0 - 3.5)	**1.2** (0 - 18)
% of RNA+	**5.55** (1 - 6.8)	**2.2** (0 - 15.7)	**4** (0 - 5.5)	**2** (0 - 17.3)
% of HMGB1+	**12.21** (0.93 - 50.7)	**9.85** (1.57 - 63.1)	**22.5** (1.09 -30.8)	**14.9** (1.6 - 66.3)
% of Citrulline+	**12.1** (3.85 - 41.97)	**11.49** (2.5 - 71.88)	**9.52** (6.78 - 16.82)	**7.33** (0 - 38.07)
% of IgG+	**3.77** (1.58 - 6.03)	**20.1** (6.19 - 71.2)	**4.51** (0.82 - 5.77)	**29.7** (9.52 - 93)
% of IgM+	**16.7** (10.4 - 39.7)	**25.2** (2.13 - 79.4)	**35.9** (25.4 - 58.9)	**40.4** (4.25 - 91.8)

*__Median__ (minimun-maximum range).

RMP and LMP were defined as <6.05% MP-IgG+

RMP-IC were defined as >28.45% MP-IgG+

LMP-IC were defined as >38.85% MP-IgG+

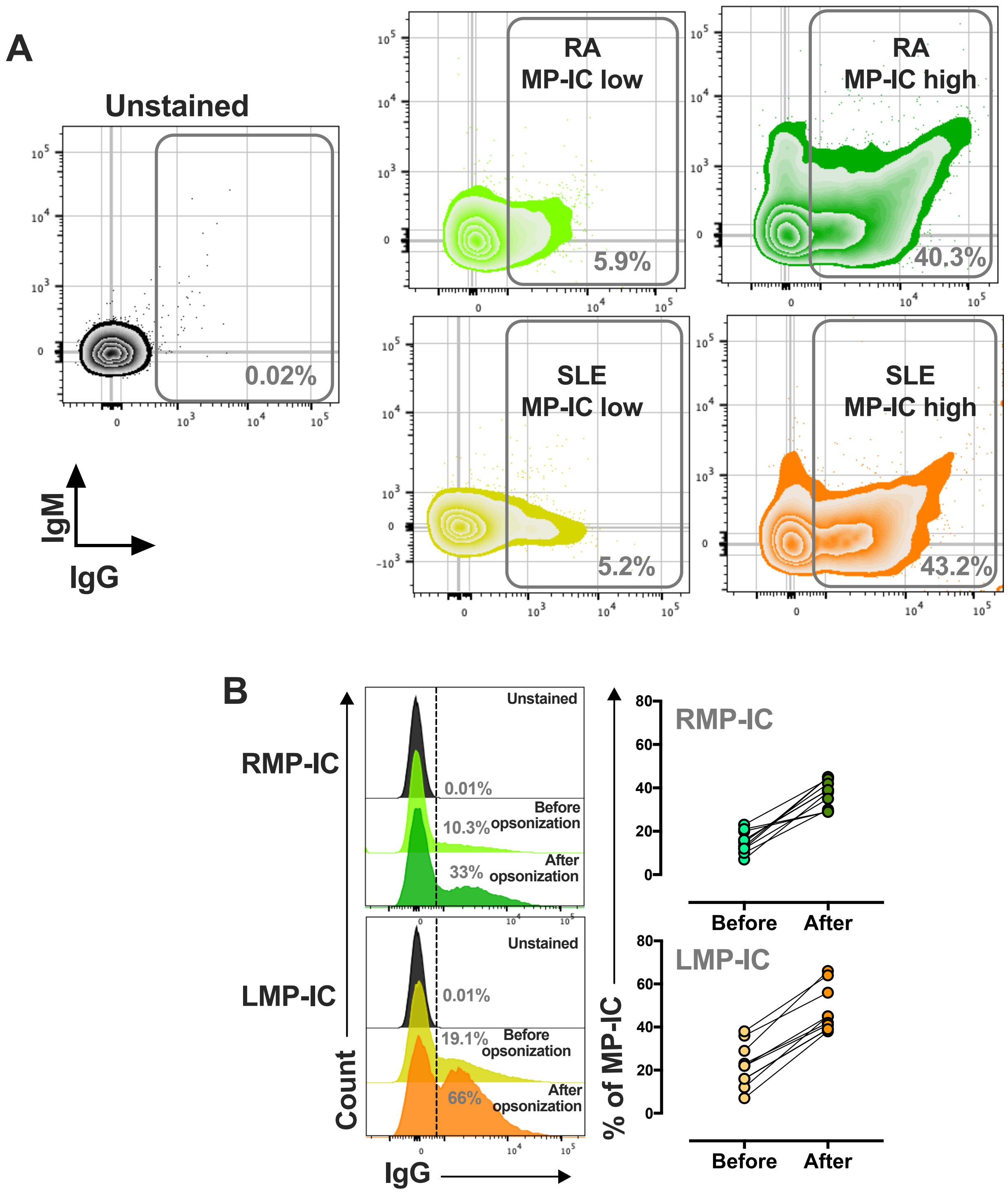

Supplementary Figure 1. MP-IC from RA and SLE patients. A. Representative density plots that show IgM and IgG content on MP from patients with RA and SLE that were included in the MP batches; *ex vivo* unstained and stained MPs with specific *F*(*ab*)2 fractions against IgG and IgM are shown before their storage at -70 °C. These AR and SLE patients belong to previously published cohorts by our group (Burbano et al, Front. Immunol. 9, 2018 and Burbano et al Sci. Rep. 8, 2018). **B**. Because the frequency of MP-IC decreased after these vesicles were thaw, *in vitro* opsonization of MPs with IgG from patients was performed.

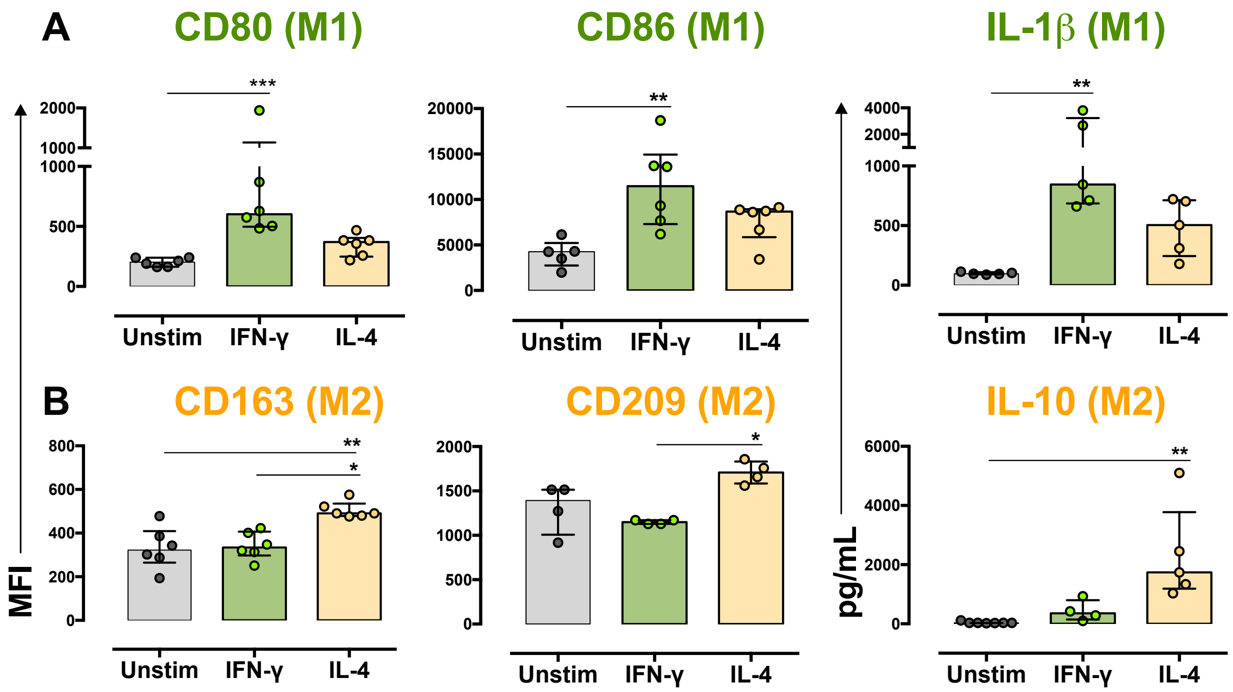

Supplementary Figure 2. IFN-γ and IL-4 induce M1 and M2 markers, respectively, on MDMs from HCs. A. The MFI of markers associated with a M1 profile in MDMs from HCs cultured without stimuli (unstimulated) or with IFN-γ or IL-4 for 6 h (n = 6). **B.** The MFI of markers associated with M2 polarisation in MDMs from HCs cultured without stimuli (unstimulated) or with IFN-γ or IL-4 for 6 h (n = 6). Comparisons among the groups were performed using the Kruskal–Wallis test and Dunn's *post-hoc* test.

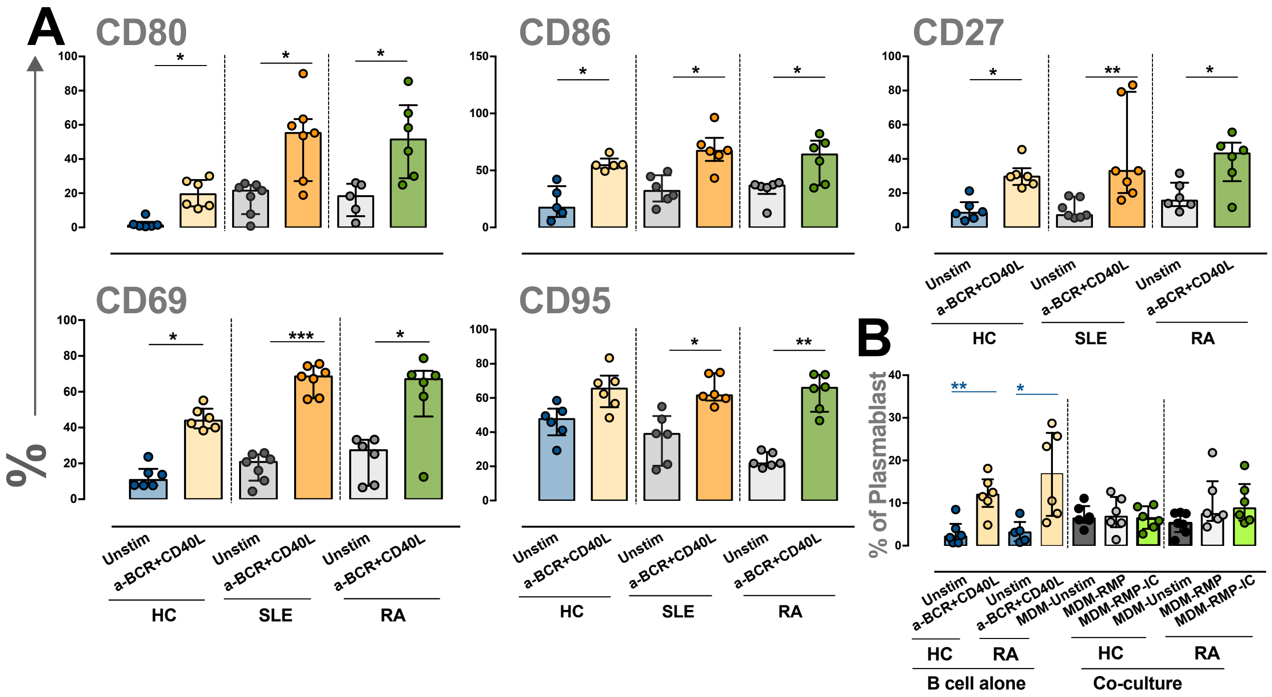

Supplementary Figure 3. B cells from patients with SLE and RA and HCs respond to conventional stimuli. **A**. The frequency of CD80, CD86, CD69 and CD95 in B cells from patients with SLE and RA and HCs (n = 6, each) that were cultured alone (Unstim, complete medium) or stimulated with anti-BCR plus CD40L. Comparisons among the groups were performed using the Kruskal–Wallis test and Dunn's *post-hoc* test. **B**. The frequency of plasmablasts in B cells cultured alone (Unstim, complete medium) or stimulated with anti-BCR + CD40L and in B cells co-cultured with MDMs differentiated without (Unstim, complete medium) or with RMPs or RMP-ICs from patients with RA (n = 7) and HCs (n = 6). Comparisons among the groups were performed using ANOVA II and the Bonferroni *post-hoc* test.

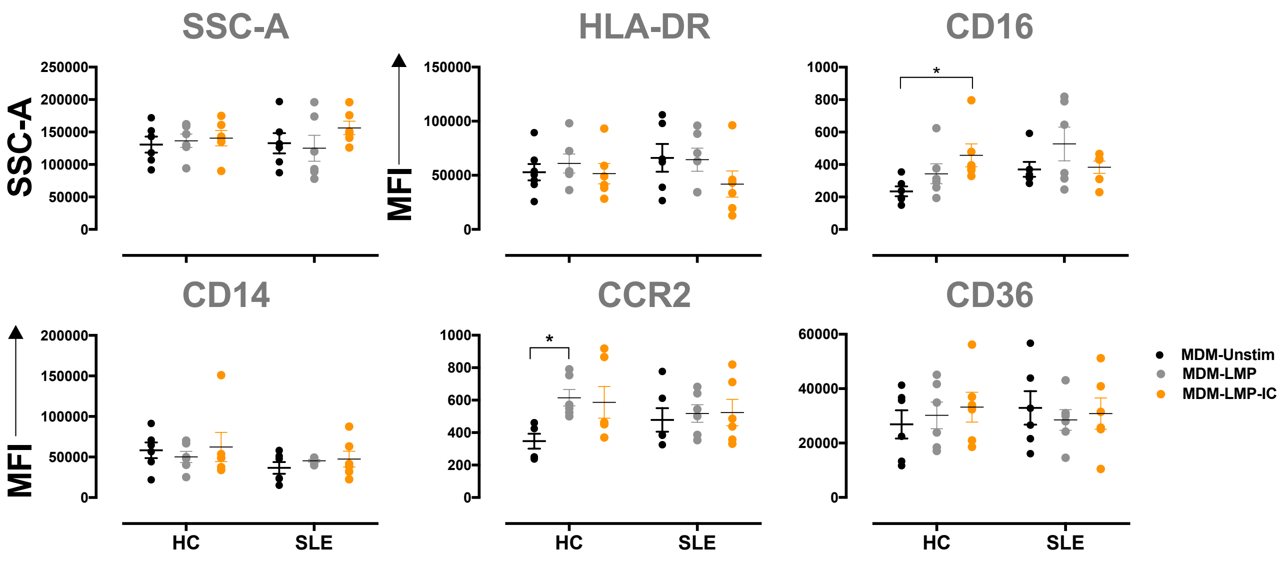

Supplementary Figure 4. MPs and MP-ICs from patients with SLE do not alter the expression of markers for MDM differentiation in macrophages from patients with SLE. The MFI of markers associated with the differentiation of mononuclear phagocytes, SSC-A, HLA-DR, CD16, CCR2 and CD36, on differentiated MDMs without (Unstimulated: Unstim, black dots) or with LMPs (dark grey dots) or LMP-ICs (orange dots) from HCs (n = 6) and patients with SLE (n = 6). Comparisons among the groups were performed using ANOVA II and the Bonferroni *post-hoc* test.

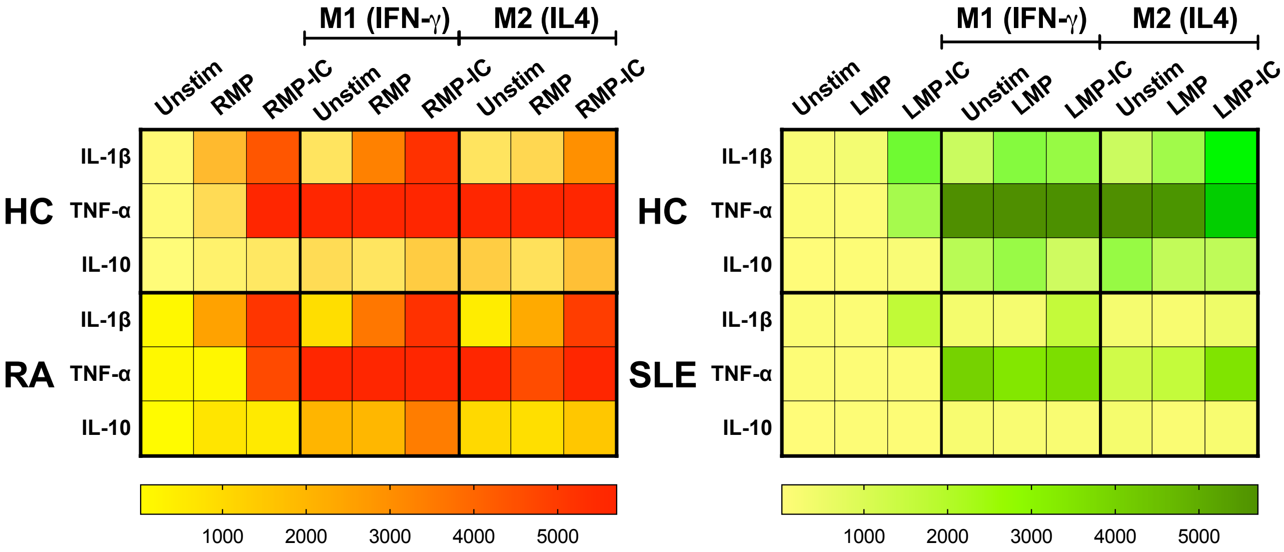

Supplementary Figure 5. RMP-ICs induce a more prominent differentiation to a M1-like profile than LMP-ICs in MDMs from HCs and patients with RA. **A** Heat map of median values of cytokine levels in the supernatants of MDMs from patients with RA and SLE and HCs that were differentiated without or with MPs or MP-ICs along with 6 h of IFN-γ or IL-4 treatment.
